# AI Model for Textile Materials Identification Using Hyperspectral Data

**DOI:** 10.3390/jimaging12060226

**Published:** 2026-05-27

**Authors:** Fariborz Eghtedari, Leszek Pecyna, Rhys Evans

**Affiliations:** The Manufacturing Technology Centre, Coventry CV7 9JU, UK; fariborz.eghtedari@the-mtc.org (F.E.);

**Keywords:** textile recycling, hyperspectral system, spectral signature, AI model, convolutional neural network, carbon-black dye, machine learning, near-infrared spectroscopy

## Abstract

Efficient textile recycling depends on accurate identification of fibre types and compositions to support high-value material recovery and automated sorting. Existing commercial systems based on near-infrared (NIR) spectroscopy offer robust performance, but their model architectures and development methods are proprietary, and they often struggle to detect materials when carbon-black (graphite-based) dyes suppress the spectral signatures. This paper presents a hyperspectral imaging approach for textile fibre identification, combined with an artificial intelligence model capable of detecting cotton, polyester, elastane, and regions affected by carbon-black dye. Sixty-five textile samples were laboratory-verified to determine constituent materials and compositions, with 52 used in model development and testing. A semi-automatic algorithm detected textile boundaries and sampled 100 spectral patches per image. For materials exhibiting two distinct spectral signatures, typically due to carbon-black dye regions, 100 samples were collected for each signature, producing a database of 6500 spectra. A convolutional neural network model was trained using these signatures to predict fibre composition and identify any regions with carbon-black dye. The system achieved mean absolute errors below 2.1% for cotton, polyester, and elastane. A spatial clustering step groups pixels with similar spectra prior to detection, enabling region-wise material identification and allowing the model to classify clusters likely affected by carbon-black dye. This approach demonstrates high precision in fibre identification and reliable detection of carbon-black regions, highlighting its suitability for real-world textile analysis workflows.

## 1. Introduction

Textile circularity relies on delivering accurately sorted fibre materials (feedstock) to recycling processes at an industrial scale. Yet, globally, only a small fraction of post-consumer textiles are transformed back into new fibres, largely due to limits in automated identification and sorting of mixed, dyed, and blended materials [1]. In practice, near-infrared (NIR) spectroscopy and hyperspectral imaging (HSI) are the leading non-tactile sensing approaches: NIR/HSI have proved robust in plastics [2,3,4,5] and, increasingly, in textiles where characteristic absorption bands allow differentiation between cotton, polyester, viscose, wool and others [6,7,8,9]. Despite this progress, persistent challenges remain:

- Carbon-black dyes obscuring underlying spectral signatures [10,11]: This problem is well-known in plastic recycling, where some plastic recycling industries are moving towards more expensive mid-infrared spectrum devices [7,12] for improved detection, as longer wavelengths are more successful in detecting carbon.
- Elastane in blends [7]: Even small elastane fractions can determine whether a textile is suitable for mechanical or chemical recycling; accurate identification of elastane (including low-percentage content) is critical for ensuring that materials are routed to the appropriate recycling pathways [1].
- The need for spatially resolved decisions across heterogeneous garments rather than single-point measurements.

Recent work indicates that pairing HSI with machine learning—particularly convolutional neural networks (CNNs)—can improve textile classification accuracy when data sampling and pre-processing steps are carefully designed [8,13].

In the UK, the Automatic Sorting for Circular Textiles (ACT UK) programme has developed a national blueprint for Advanced Textile-Sorting and Pre-Processing (ATSP) facilities to supply consistent, high-quality feedstock for mechanical and chemical recyclers [1]. As the report highlights, the establishment of ATSP facilities requires a detailed understanding of the performance, limitations, and integration potential of emerging sensing and sorting technologies. To support these objectives, ACT UK commissioned a series of comparative technology trials—including hyperspectral imaging, NIR single-point sensors, RGB-based approaches, and mechanical-sorting concepts—to evaluate their suitability for large-scale automated textile sorting. The hyperspectral AI trials presented in this paper were conducted as part of this broader programme, using verified textile samples and controlled acquisition conditions.

Despite the emergence of commercial NIR/HSI solutions for textile sorting, publicly documented model designs, training datasets, and evaluation protocols remain scarce, making independent validation and fair comparison difficult. A further complication is data reliability: garment labels are frequently inaccurate or incomplete, undermining both ground truth and model training, and contributing to inconsistent performance claims across studies and deployments [1,14]. In our training dataset, a composition error of over 3% between the fabric labels and laboratory-verified values was seen in over 30% of the samples, further underscoring this issue.

The objective of this work is to assess the capabilities and limitations of a transparent HSI–CNN with an emphasis on known challenges: carbon-black dyes, low-percentage elastane, and spatially heterogeneous garments. The main contributions of this study include the following:

- Construction of a spectral database via semi-automatic sampling using lab-verified textiles;
- Introduction of a process of spatial clustering prior to region classification, allowing accurate identification of regions characterised by different spectra, including regions containing carbon-black dye;
- Identification and exclusion of carbon-black-dye-affected clusters so that composition estimation is restricted to unmasked regions.

While this work is not a benchmarking standard, it is designed to reduce ambiguity by (i) disclosing the sampling, clustering, and masking steps, and (ii) using independently sourced test pieces in addition to original development samples.

The remainder of this paper is structured as follows: The next section reviews related work on NIR/HSI sensing for textiles and neural-network-based classification. Following that, the materials and methodology are presented, including a verified textile sample list, the hyperspectral acquisition setup, semi-automatic sampling, spatial clustering, and the neural-network-training and -testing procedures. The Results Section presents fibre composition estimates, detection of carbon-black regions and additional evaluation of elastane content. We, then, outline the model usage and detection pipeline, before concluding with final remarks.

## 2. Related Work

### 2.1. Hyperspectral/NIR Sensing for Textile Identification

Near-infrared (NIR) spectroscopy and hyperspectral imaging (HSI) have become established in materials identification for non-destructive textile identification due to their wavelength operation range of 780 nm to 1700 nm, which is effective in identifying specific chemical groups existing in fibres (e.g., C-H, O-H, and C-O in cellulose) [6]. This makes the technology suitable for the differentiation of various textile fibre materials [6,7,8,9].

Industrial system reviews and trials cited in the ACT UK programme similarly report that while commercial NIR/HSI scanners can separate common mono-materials at scale, accuracy degrades for mix-blended materials, for elastane at low percentages, and in black/grey regions due to dye masking. Mid-infrared extensions and data-driven modelling are being explored to mitigate these gaps [1]. Our previous work with an area scan NIR camera + band-pass filters demonstrated an illumination-robust metric for distinguishing between cotton and polyester. It showed that carbon-black dyed regions can be identified, motivating region-wise exclusion or specialised handling before fibre classification [15].

### 2.2. Machine Learning and Neural Networks for Spectral Textile Classification

With access to high-dimensional spectral inputs, machine learning approaches, particularly convolutional neural networks (CNNs), have demonstrated strong performance in hyperspectral image classification tasks by learning distinguishable spectral–spatial features directly from the data. Such approaches have been successfully applied across a range of material identification problems, including plastics and agricultural products [3,5,16].

When applied to textile classification, CNN-based models have shown improved performance over classical analytical methods by learning distinctive features directly from NIR/HSI signatures [8,13]. Recent studies, applying deep learning to textile identification and sorting, report strong accuracies for the most common fibre types. However, these deep learning approaches do not address the earlier-mentioned limitations: they do not handle carbon-black dye masking, which can suppress spectral information, nor do they consider textiles with multiple distinct spectral signatures arising from prints, coatings, dye-affected regions or material types.

A related approach is presented in [17], where hyperspectral data are combined with a compact neural network to enable rapid detection and localisation of contamination within cotton. Their work highlights the effectiveness of band selection strategies and lightweight models for spatial detection tasks. However, these approaches focus primarily on localisation and classification of foreign materials. In contrast, the present study focuses on per-signature fibre composition estimation and carbon-black detection, with spatial variability handled via a separate clustering stage rather than being learned directly within the network.

As noted in the Introduction, despite the existence of commercial NIR/HSI-based sorting solutions, publicly documented model architectures, training datasets, and evaluation protocols remain scarce, limiting the comparability and independent validation of reported performances across vendors and studies. Transparent, reproducible machine learning pipelines remain essential for assessing the true capabilities and limitations of NIR/HSI-based textile identification.

Building on prior NIR/HSI–deep learning research and our earlier NIR imaging work, the present study contributes a fully documented HSI+CNN workflow that

1. Constructs a lab-verified spectral database via semi-automatic sampling;
2. Conducts spatial clustering prior to detection to produce region-wise composition estimates;
3. Explicitly identifies carbon-black-affected clusters so that composition estimation is restricted to unmasked regions.

## 3. Materials and Methods

### 3.1. Textile Test Samples

Textile samples of known fibre materials and compositions were required to establish the ground-truth data for training the AI model. In addition, further samples were needed to investigate the performance of the trained model at the inference stage.

The majority of textiles are made of polyester, cotton or a mixture of the two, with varying blend proportions. Small amounts of elastane may also be included in some textiles to provide stretch. It was reported that in 2024, around 59% of world production was polyester [18], and around 19% was cotton [19]. The bulk of textiles in production is made out of cotton, polyester or a mixture of the two. To demonstrate the feasibility of a hyperspectral-based fibre identification system, this study focused on textiles composed of 100% polyester, 100% cotton, or polyester–cotton blends, with some samples containing elastane.

Our research showed that garment labels are not a reliable indicator of the composition of the fibres in textiles and, in most cases, can be inaccurate. As such, Bureau Veritas (Birmingham, UK), a world leader in testing, inspection and certification, was approached and asked to provide verified textile samples to create a pool of materials of known fibre materials and compositions. Forty-six textile samples of varying compositions of polyester, cotton and elastane were sourced and tested to get their true material compositions.

Table 1 shows the details of the 46 samples that were laboratory-tested. The testing methods used to determine the materials’ fibre compositions were not capable of identifying the presence of carbon-black dye. As can be seen, there were discrepancies in the vendors’ claimed compositions of the textiles and laboratory test results. Table 2 shows the error ranges for the percentage of the population with respect to the 46 tested samples.

An independent test set was provided by a major UK retailer. The claimed compositions and laboratory test results of the true compositions of the samples are shown in Table 3. These samples were not used in model development (training or validation) but were used to provide an additional measure for the performance of the model.

### 3.2. Hyperspectral System Setup and Data Acquisition

The hyperspectral camera used in this trial was a Specim FX17 (Specim, Oulu, Finland). It has a sensitivity range of 900 to 1700 nm, providing a spatial resolution of 640 pixels with 224 spectral bands per pixel.

For visualisation purposes, hyperspectral images shown throughout this study are displayed using a false-colour RGB representation, where three selected spectral bands from the 900–1700 nm range are mapped to the red, green, and blue channels. These colours, therefore, do not correspond to visible wavelengths but instead reflect differences in spectral signatures between regions.

The FX-17 operates as a line scanner, requiring relative movement between the camera and the object to build a two-dimensional image. This system is well-suited to industrial applications where the camera is fixed, and objects are placed on a moving conveyor system.

**Figure 1:**
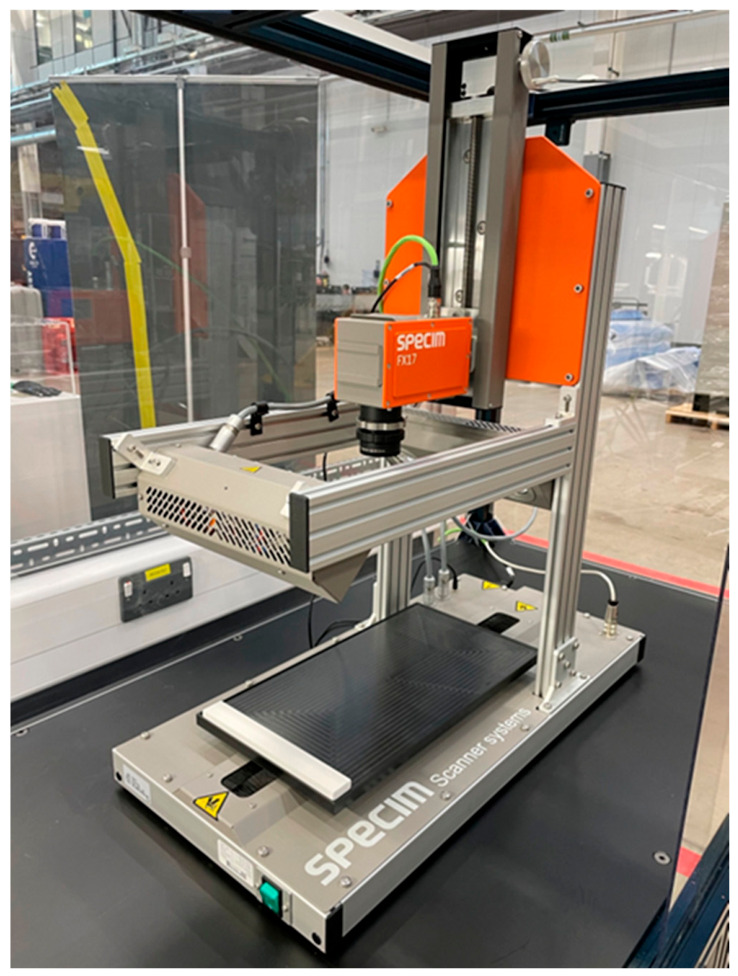
Hyperspectral system set up.

In our trials, as shown in Figure 1, the camera was mounted within the LabScanner, which includes a linear stage and two sets of fan-cooled halogen lights. Halogen lights offer extensive coverage of light wavelengths in the NIR range. The linear stage is software-controlled, with its travel speed synchronised to the camera scan rate to allow the software to reconstruct an image of the object under inspection.

The test sequence followed for data acquisition was as follows:

1. Place test textile samples individually on the linear stage;
2. Move the linear stage under software control over a pre-specified length;
3. Scan the moving sample with the hyperspectral camera;
4. At the end of the scan, save the acquired data for later analysis.

### 3.3. Data Pre-Processing: Region Extraction, Sampling, and Clustering

Following hyperspectral acquisition, each textile image underwent a pre-processing workflow designed to extract representative spectral signatures from the fabric surface while accounting for spatial variability and the potential presence of carbon-black-dyed regions. Prior to further processing, the hyperspectral data were calibrated using standard dark and white reference images. For each acquisition, a dark reference (sensor response without illumination) and a white reference (uniform reflectance target) were recorded. The raw hyperspectral data were corrected using these references to obtain relative reflectance values, reducing the influence of sensor noise and illumination variability.

#### 3.3.1. Region Extraction

For each hyperspectral image, the boundaries of the textile sample were automatically detected to exclude the background and any non-textile elements. Only pixels within the included regions were retained for subsequent sampling. This ensured that the extracted spectra originated exclusively from the textile surface and were not affected by the conveyor, supporting hardware, or regions outside of the image boundaries.

**Figure 2:**
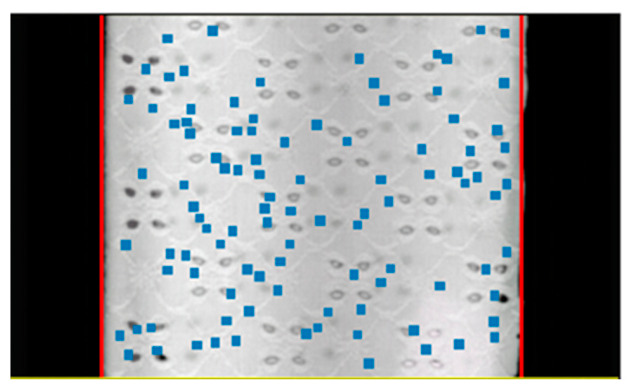
Automatic collection of 100 spectral samples (false-colour representation).

Boundary detection was performed by treating each pixel as a 224-dimensional spectral vector and computing the average spectrum of the entire image. The algorithm then searched the top/bottom rows and left/right columns for locations where the Euclidean distance between the pixel spectrum and the average spectrum exceeded a predefined threshold. These points defined the cropping boundaries, enabling automatic isolation of the textile area. This approach relies on the assumption that the textile occupies the majority of the field of view, and the average spectrum, therefore, approximates the textile’s dominant signature. A simple user interface was implemented to allow the operator to confirm the automatically extracted region or to manually adjust the cropping if necessary. The cropped region is marked with red lines in Figure 2.

#### 3.3.2. Patch-Based Spectral Sampling

**Figure 3:**
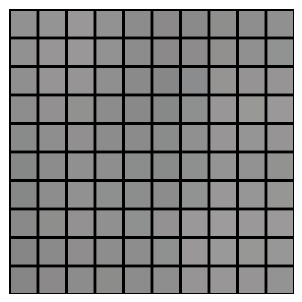
Illustration of a sampled 10-by-10-pixel patch used to generate a single spectral signature. Each pixel is visualised as an enlarged block for clarity.

To generate a robust training dataset, a semi-automatic sampling algorithm selected multiple small spatial patches at random locations within the extracted textile region. Each patch contained 100 pixels (10 by 10 patch; see Figure 3), and the mean spectrum of these pixels was computed to produce a stable hyperspectral signature. For each textile sample, 100 spectral patches were generated in this manner. The patch-based strategy reduced the influence of pixel-level noise and local spectral variability, producing more stable hyperspectral signatures while ensuring that the training dataset captured within-fabric variability while remaining computationally efficient (see the blue rectangles in Figure 2; they correspond to 10-by-10-pixel patches).

#### 3.3.3. Handling Textiles with Multiple Spectral Signatures

**Figure 4:**
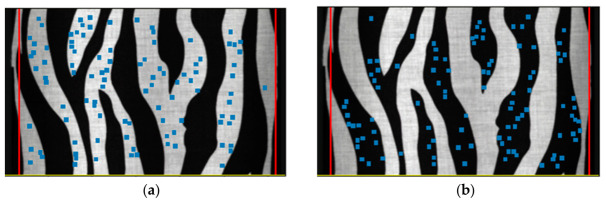
Collection of two sets of data from textile piece with black patterns (false-colour representation): (**a**) sampling from the region containing material not covered by carbon-black dye; (**b**) sampling from the region covered by carbon-black dye.

Before sampling, a clustering algorithm was applied to identify regions exhibiting distinct spectral behaviour. Some textiles—particularly those containing carbon-black dye or strong printed patterns—displayed two distinct spectral signatures within the same image. To correctly represent this variability, the algorithm identified clusters of pixels whose spectra deviated significantly from the dominant signature. To implement this, first, the mean spectrum was calculated for the cropped region (224-dimensional vector), and then the Euclidean distance was calculated between the mean vector and all pixels in the cropped region. Following that, a threshold was applied. The threshold was dynamically adjusted (the user increased or decreased the threshold during data collection and observed the changes to the clusters). For each spectral cluster, 100 patches were collected, ensuring that both unmasked (material-revealing) and masked (dye-affected) regions were included in the dataset. (Please see the two sets of patches collected from these two clusters illustrated in Figure 4.)

**Figure 5:**
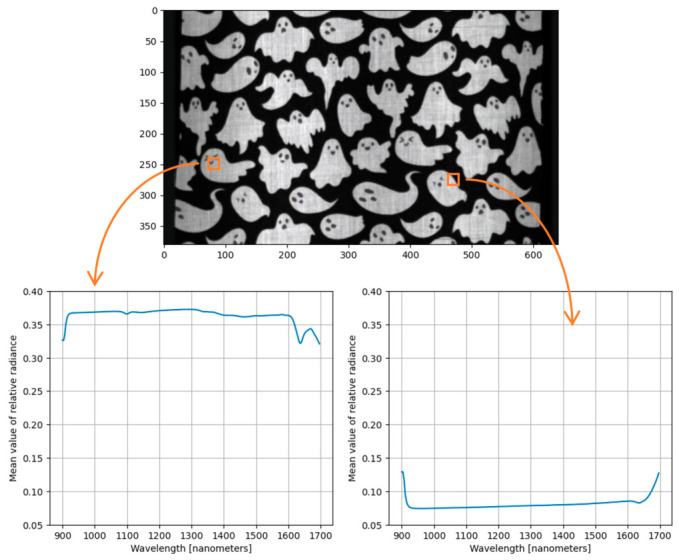
The difference in spectrum in region with no carbon-black dye and region containing the dye (false-colour representation).

Through patch-based sampling and spectral clustering, this pre-processing pipeline ensured that the model was trained on stable and representative spectral signatures. Figure 5 illustrates the difference in the spectrum between two different regions of the image. A similar mechanism was later reused to detect and exclude carbon-black-masked regions during inference, preventing these from adversely affecting fibre composition prediction (see Section 4).

### 3.4. Neural Network Model Architecture

The identification model operates on 1D hyperspectral signatures (224 bands per input) and predicts two output types:

1. Fibre composition (cotton, polyester, and elastane; continuous outputs), with 3 output neurons;
2. The presence of carbon-black dye (binary output), with 1 output neuron.

To exploit local spectral patterns while limiting model size, a compact convolutional neural network (CNN) with a single 1D convolutional layer was adopted, followed by a lightweight fully connected block. The convolutional layer extracts band-local features (learned spectral filters), while the dense layers combine them into global representations suitable for composition regression and dye classification. The model branches at the output: one regression head (three neurons for the fibre compositions) and one classification head (one neuron for carbon-black probability). A joint, two-component training objective enables multi-task learning, encouraging shared representations that benefit both tasks.

**Figure 6:**
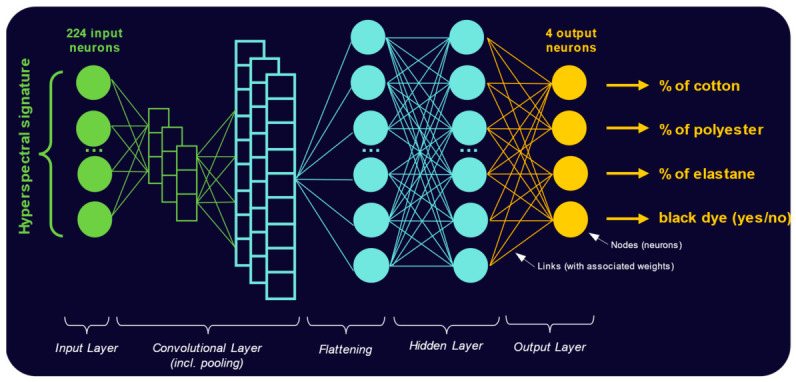
Simplified depiction of artificial neural network used.

The 1D convolution layer operates along the spectral axis, treating each hyperspectral signature as a one-dimensional sequence of 224 wavelength values. The convolution, therefore, captures local relationships between neighbouring wavelength bands, enabling the model to learn short-range spectral patterns. A compact architecture was favoured to reduce the overfitting risk, given the dataset size, and to keep inference latency low for potential deployment. The multi-head design reflects the operational workflow: composition is only trusted in non-carbon-black regions, so learning to detect dye explicitly complements composition estimation. The design of the network is illustrated in Figure 6.

Outputs

- Regression head: $y_{fib}\in R^{3}$ for cotton, polyester, and elastane, interpreted as fractional fibre contributions.
- Classification head: $y_{clf}\in \left[0,\;1\right],$ indicating the probability that the input spectral signature is affected by carbon-black dye.

Hyperparameters

A hyperparameter search was performed to optimise the model’s performance; the following network hyperparameters were found:

- Batch size: 16;
- Number of filters in convolution layer: 3;
- Convolution layer kernel size: 3;
- Hidden layer size: 36;
- Initial learning rate: 0.001.

This compact network contains approximately 12k trainable parameters, and its inference time is in the order of sub-milliseconds on standard CPU hardware. The model was implemented in Python (version 3.12) using the TensorFlow [20] (version 2.19) package. The Adam [21,22] optimiser was used for model training.

### 3.5. Neural Network Training and Validation Procedures

Training uses the patch-based signatures produced by pre-processing. Signatures are grouped by textile sample, and the dataset is split into training and validation subsets such that validation signatures come from samples not used in gradient updates. The validation set is used for model selection and hyperparameter tuning. The results reported in Section 4 distinguish between (i) the main validation results and (ii) the independent test set acquired later from an independent source, which was not used in training or model tuning.

Normalisation and batching: Each hyperspectral signature is normalised prior to training to improve numerical stability and to reduce sensitivity to illumination differences inherent to imaging hardware. Specifically, min–max scaling is applied, such that all input values are mapped to the range [0, 1], using global minimum and maximum values computed across the dataset. Mini-batches are randomly sampled from the training subset, preserving the mixture of clusters (unmasked vs. masked (carbon-black-affected)) to stabilise the multi-task loss during optimisation.

Multi-task loss: The model is trained with a two-component objective that jointly optimises composition regression and dye classification:

(1) $$L=w_{fib}L_{fib}+w_{clf}L_{clf}$$

where $L_{fib}$ measures the discrepancy between predicted and ground-truth fibre percentages (in this case, the mean-squared error was utilised), and $L_{clf}$ is a binary classification loss (cross-entropy loss was used) for the carbon-black label. The weights $w_{fib}$ and $w_{clf}$ balance the tasks during training, empirically chosen as $w_{fib}=0.2$ and $w_{clf}=0.2$. This formulation encourages shared spectral features that are useful for both quantitative composition estimation and dye-masking detection.

**Figure 7:**
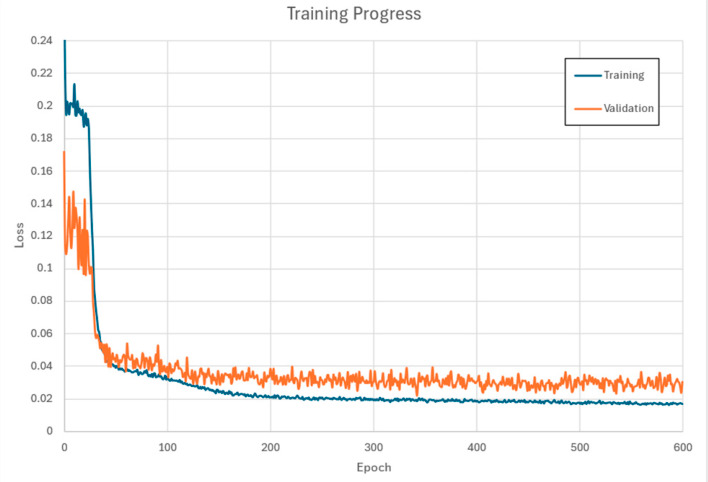
An example of fibre loss value change over the training time, for both training and validation sets. The model was trained for 600 epochs.

Training proceeds for multiple epochs while monitoring the validation loss and associated metrics (e.g., composition error and dye classification accuracy). Curves of training/validation accuracy over epochs were used to diagnose under/overfitting and to select final hyperparameters (see Section 4 for performance summaries). An example learning trajectory over 600 epochs is shown in the training curves (Figure 7).

#### Training–Validation Split

Because each textile piece yields a large number of similar spectral samples (typically 100 patches per spectral mode), random sample-level splitting would generate near-duplicate signatures between the training and validation sets, artificially inflating validation performance. To avoid this, the training–validation split was performed at the textile sample (image) level, ensuring that all spectral samples originating from the same physical fabric were assigned entirely to either the training or validation subset. As a result, the model was evaluated only on spectral signatures originating from textile pieces it had never seen before, providing a more realistic assessment of generalisation performance.

To maintain a balanced representation of fibre types across splits, textile pieces were first sorted by ground-truth fibre fraction, after which every third piece (≈33%) was assigned to the validation set, with the remaining pieces (≈67%) used for training. This approach preserved the fibre materials distribution between sets.

As illustrated in Table 1, 46 laboratory textile pieces were used. Due to errors during data collection and processing, three pieces were excluded. Thirteen scanned textile samples contained some regions affected by carbon-black dye. This led to 56 different regions and a total of 5600 spectral samples. The split described above was applied, and 19 textile samples (1900 spectral samples) were used for validation. In the process of model hyperparameter search, this split was done three-fold each time using different parts of the dataset as validation. The average values were used to compare model results between different architectures and select final hyperparameters (see Section 3.4). Apart from training and validation datasets, an independent test set was created from 19 samples listed in Table 3. Only relevant textile pieces containing a mix of cotton, polyester and elastane were selected (eight textile pieces, one containing a carbon-black-dyed region), leading to 900 spectral samples. Section 4.1 outlines more details on the use of this dataset.

### 3.6. Performance Metrics

#### 3.6.1. Fibre Identification—Mean Absolute Error

To evaluate the model’s ability to estimate fibre composition and detect carbon-black-affected regions, several quantitative metrics were used. For the regression task, performance was measured using the mean absolute error (MAE) between the predicted and ground-truth fibre fractions for cotton, polyester, and elastane. The MAE was computed both per fibre type and as an average across all three fibres, allowing a detailed assessment of model behaviour.

#### 3.6.2. Carbon-Black Dye Detection—Binary Accuracy

Because carbon-black dye can mask the underlying spectral signal, the model in parallel performs classification. This is reflected in the last output/neuron of the model. The model outputs a probability $p_{\mathrm{black}}\in [0,\;1]$ per sample signature. During evaluation, TensorFlow’s binary accuracy metric was used, which applies a fixed decision threshold of 0.5 to convert probabilities to labels (black if $p_{\mathrm{black}}\geq 0.5$; non-black otherwise). Binary accuracy is then computed as the proportion of correctly classified clusters across the evaluation split.

#### 3.6.3. Elastane Contribution—Categorical Accuracy

Because the elastane contents in the verified samples were small (≤10%), absolute errors in regression could translate into large relative deviations. Moreover, when elastane is absent, but the model predicts a non-zero fraction, the relative error is formally unbounded. To provide a more informative assessment, an additional evaluation was performed. Elastane outcomes were evaluated as a three-class classification problem with the following categories:

- No elastane;
- 1–3% elastane;
- >3% elastane.

Model outputs were mapped onto these categories and scored using categorical accuracy computed over sample-level predictions.

## 4. Results

This section presents the performance of the proposed HSI–CNN model across both the development dataset (training/validation splits) and an independent set of additional textile samples not used during model development. As the predictions from both groups of samples were combined, the independent test set evaluation is reported first, followed by detailed analyses of fibre composition accuracy, carbon-black dye classification, and elastane content evaluation.

### 4.1. Independent Test Set Evaluation on Additional Textile Samples

An independent test set was provided by a major UK retailer. These samples were not used in model development (training or validation) and were selected to contain only the three fibre materials targeted by the model (cotton, polyester, and elastane), yielding eight textile pieces in total. One piece exhibited pixels with a distinct spectral behaviour (two clusters were identified), resulting in nine test regions overall (IDs: TA04, TA08, TA09, TA11, TA15, TA16, TA17, TA19, and TA19B). Table 4 summarises the per-region ground truth and the corresponding average model outputs for the independent set.

Across the nine test pieces, the model achieved the following mean absolute error complements (“precision”) on non-carbon-black-dyed regions: 2.9% for cotton, 3.4% for polyester, and 2% for elastane, with a 2.8% average. These values are in line with the validation set performance reported in Section 4.2, suggesting reasonable generalisation to unseen samples acquired under comparable conditions.

**Figure 8:**
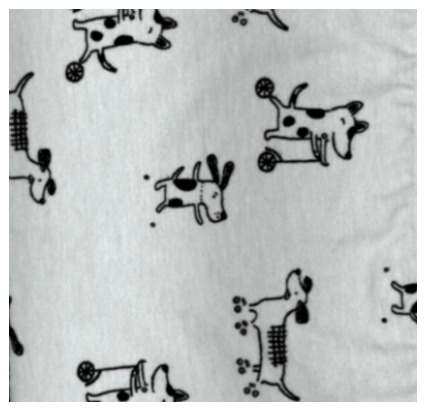
Image of the textile piece (false-colour representation) containing a carbon-black-dyed region (TA19B).

One of the nine independent test pieces (TA19B) included a region dyed with carbon-black. The model correctly identified this region. However, because only a single carbon-black-dyed instance was present in the independent test set, standalone evaluation of carbon-black dye classification performance on the test data is not statistically meaningful. Accordingly, carbon-black dye detection performance is reported using the development data (validation set) in Section 4.3. As expected, the fibre composition predictions for the carbon-black-dyed region in TA19B were highly inaccurate, consistent with the model design, which does not estimate composition in dye-affected areas. For clarity, an image of a textile sample from the additional test set, which contains printed patterns using carbon-black dye, is shown in Figure 8.

### 4.2. Fibre Composition Detection Precision (Absolute Errors)

The MAE for cotton, polyester, and elastane was reported on the development (validation) dataset, following the evaluation process presented in Section 3.6. Because carbon-black dye can mask the underlying spectral signal, the analysis was separated into non-black, carbon-black-dyed regions and overall. The numerical results are given in Table 5. In summary, non-black regions yielded low errors (average MAE ≈ 1.8%), whereas carbon-black-dyed regions exhibited higher errors due to spectral masking (average MAE ≈ 4.9%).

**Figure 9:**
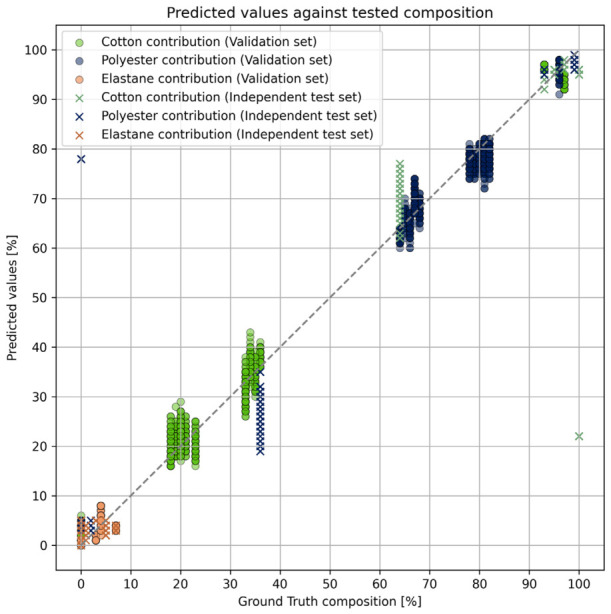
Predicted vs. verified fibre composition for the development (circles) and independent test (crosses) signatures. The grey dashed diagonal indicates perfect agreement. Colour coding by fibre type: cotton (dark blue), polyester (green), and elastane (orange). Points closer to the diagonal correspond to lower absolute error.

Figure 9 visualises the predicted fibre fractions against verified ground-truth values for both the validation signatures and the independent test signatures (marked distinctly; see legend). The grey dashed diagonal line represents perfect agreement; points closer to this line correspond to lower absolute error. Each fibre type (cotton, polyester, and elastane) is colour-coded for clarity. Regions containing carbon-black dye were excluded from this plot. The results sometimes appear as vertical stacks of points; this occurs when multiple predictions originate from the same textile piece with the same ground-truth composition, so variation is only in the predicted values.

For elastane, the absolute errors remained small; however, because elastane percentages within all samples were <10%, even a ~1–2% MAE could lead to a large relative deviation. This is further addressed in Section 4.4.

### 4.3. Carbon-Black Dye Classification Accuracy

Detection of carbon-black-affected regions was achieved by using the binary accuracy metric described in Section 3.6. Across all sampled signatures throughout all regions/textile pieces in the validation set, the model achieved a carbon-black dye classification accuracy of 96.4%. This indicates that the model reliably distinguishes dye-masked regions from unmasked textile areas, ensuring that dye-affected clusters can be excluded prior to fibre composition estimation. As noted previously, only a single carbon-black-dyed instance was present in the independent test set (TA19B), and this region was correctly identified.

### 4.4. Elastane Content Evaluation (Absolute and Relative Performance)

**Figure 10:**
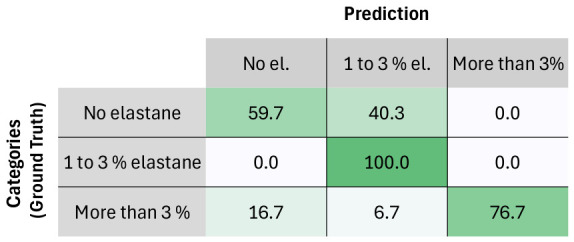
Confusion matrix for the elastane three-category evaluation on the validation set. The green colour shade relates to the values in the matrix.

Elastane performance was additionally assessed using the three-category evaluation scheme described in Section 3.6. On the validation set, the model achieved an overall elastane-category accuracy of 64.9%. The corresponding confusion matrix (Figure 10) shows that the dominant error mode occurs for samples containing no elastane, where 40.3% are misclassified into the 1–3% category. This highlights the difficulty of distinguishing very small elastane fractions in blended textiles. This behaviour is partially due to the regression-based nature of the model, where small non-zero predictions can result in assignment to the low-elastane (1–3%) category. Additionally, very low elastane concentrations produce weak spectral signatures due to a number of factors, including the small thickness of the elastane when stretched in the weaving or knitting process, the limited resolution of the imaging cameras compared with the thickness of the elastane, and the fact that elastane present in the textile is woven within the main fibre(s) used in the body of the textiles; thus, a significant portion of the elastane will be hidden from the imaging sensors, making them difficult to distinguish from elastane-free samples. This limitation is consistent with known challenges in hyperspectral textile analysis, where elastane may be present either as part of the fibre blend or as separate fibres with limited spectral visibility at low concentrations.

## 5. Detection Workflow in Model Execution (Inference)

**Figure 11:**
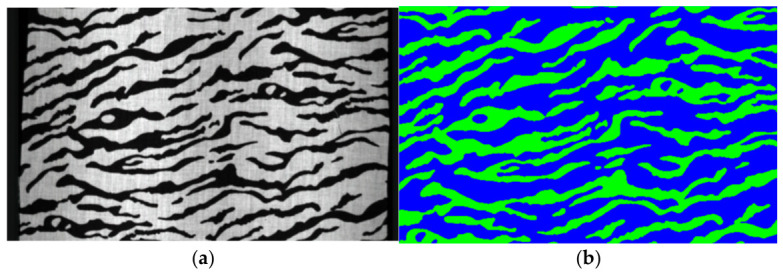
Effect of hyperspectral image clustering: (**a**) grayscale image of the textile sample; (**b**) clustered hyperspectral image with two clusters visualised by different colour masks (green and blue).

In addition to the pre-processing steps described in Section 3.3, an extended workflow was implemented for model execution, enabling region-wise fibre composition analysis and carbon-black detection directly from a full hyperspectral image. The cropped textile region was subjected to K-means clustering, where the number of clusters *n* is currently specified by the user (typically *n* = 2 or *n* = 3 depending on the expected number of distinct spectral behaviours). Each cluster grouped pixels with similar spectral signatures and produced a spatial map visualising the segmentation. From each cluster, the mean spectrum was calculated and passed to the trained model, which output both (i) the estimated fibre composition and (ii) the probability of carbon-black influence for that cluster. An example of the clustering-based segmentation is shown in Figure 11. In the current implementation, cluster selection is supported by a simple user interface that provides immediate visual feedback, allowing the user to adjust the grouping based on observed spectral variation. Automated methods for determining the optimal cluster configuration could be explored in future work.

## 6. Conclusions

The results demonstrate that hyperspectral imaging combined with a lightweight CNN model can reliably estimate the fibre composition of textiles containing cotton, polyester and elastane when region-aware spectral signatures are used. Low MAE values obtained for non-carbon-black-dyed regions confirm that the model generalises well across textiles with varied compositions and dye patterns, provided that the underlying spectral information remains observable. As expected, carbon-black dyes introduce spectral masking that leads to higher estimation errors, reinforcing the importance of identifying and excluding dye-affected clusters prior to composition analysis. The ability to isolate regions with carbon-black dye enables fibre identification even on partially masked items.

The independent test set evaluation confirms that model performance remains stable on unseen textiles acquired separately from model development. The correct identification of the only-carbon-black-dyed region in the independent set further supports the robustness of the dye detection component.

This study presents a transparent hyperspectral AI workflow for textile fibre identification using verified cotton–polyester–elastane samples. A semi-automatic sampling pipeline and region-wise clustering approach were used to construct a representative dataset to allow cluster-level material identification. The resulting CNN model achieved low MAE values for composition estimation on both development and independent test sets (with an average of 1.8% across all fibre types for the validation set and 2.8% for the test set). The method also demonstrated high accuracy (96.4%) in detecting carbon-black-affected regions, enabling reliable exclusion of masked spectra from composition estimation.

Elastane detection remains challenging due to its typically low contribution and limited spectral visibility in the NIR/SWIR range. Detecting low-percentage elastane remains operationally important as even a minor elastane presence can influence sorting decisions and determine the suitability of a textile for specific mechanical or chemical recycling pathways. The three-category elastane assessment provides additional interpretability and offers practical classification. The results highlight that most elastane-containing samples are correctly placed within the appropriate category, although false positives at very low elastane levels remain a limitation.

Overall, the findings show that a region-aware hyperspectral pipeline can address several key challenges, including carbon-black masking. The remaining limitations include a limited number of elastane-containing blends with wider ranges of elastane proportions, and the absence of other fibre materials that may be present in real waste streams (e.g., viscose, wool, and nylon), which should be addressed in future work. Direct comparison with existing deep learning approaches was not performed, as the models reported in the literature typically address different tasks (e.g., classification or detection) and are trained on dataset-specific spectral ranges and preprocessing pipelines. A meaningful benchmark would require reimplementation and retraining of these models on a common dataset with consistent evaluation criteria. This represents an important direction for future work.

## Figures and Tables

**Table 1:** Sample list and fibre compositions (label and laboratory-tested).

Job No.	Fabric Description	Sample	Claimed Composition	Laboratory Test Results	% Error
290566	Pink Pattern Knit Fabric	1	95% Polyester; 5% Elastane	90.4% Polyester; 9.6% Elastane	4.6
290567	Pink Knitted Fabric	2	95% Polyester; 5% Elastane	97.6% Polyester; 2.4% Elastane	2.6
290568	Multi-Stripe Knitted Fabric	3	95% Polyester; 5% Elastane	95.7% Polyester; 4.3% Elastane	0.7
290569	Navy Knitted Fabric	4	95% Polyester; 5% Elastane	95.8% Polyester; 4.2% Elastane	0.8
290571	Nude Mesh Fabric	5	95% Polyester; 5% Elastane	94.7% Polyester; 5.3% Elastane	0.3
290572	Red Knitted Fabric	6	95% Polyester; 5% Elastane	95.8% Polyester; 4.2% Elastane	0.8
290573	Blue/Black Tartan Woven Fabric	7	95% Polyester; 5% Elastane	95.7% Polyester; 4.3% Elastane	0.7
290574	Turquoise Satin Woven Fabric	8	95% Polyester; 5% Elastane	96.3% Polyester; 3.7% Elastane	1.3
290575	Pink Faux Suede Knitted Fabric	9	95% Polyester; 5% Elastane	92.8% Polyester; 7.2% Elastane	2.2
290642	Black Floral Woven Fabric	10	95% Polyester; 5% Elastane	92.9% Polyester; 7.1% Elastane	2.1
290576	Tan Woven Fabric	11	95% Cotton; 5% Elastane	97.0% Cotton; 3.0% Elastane	2
290577	White/Multi-Print Woven Fabric	12	95% Cotton; 5% Elastane	96.8% Cotton; 3.2% Elastane	1.8
290578	Stone Woven Fabric	13	95% Cotton; 5% Elastane	98.0% Cotton; 2.0% Elastane	3
290579	Red Floral Woven Fabric	14	95% Cotton; 5% Elastane	96.7% Cotton; 3.3% Elastane	1.7
290580	Mustard Woven Fabric	15	95% Cotton; 5% Elastane	98.0% Cotton; 2.0% Elastane	3
290581	Deer Print Knitted Fabric	16	95% Cotton; 5% Elastane	94.2% Cotton; 5.8% Elastane	0.8
290582	Pink Love Print Knitted Fabric	17	95% Cotton; 5% Elastane	93.4% Cotton; 6.6% Elastane	1.6
290583	Navy Tractor Print Knitted Fabric	18	95% Cotton; 5% Elastane	94.1% Cotton; 5.9% Elastane	0.9
290584	Navy Floral Knitted Fabric	19	95% Cotton; 5% Elastane	92.9% Cotton; 7.1% Elastane	2.1
290643	Mustard Knitted Fabric	20	95% Cotton; 5% Elastane	96.0% Cotton; 4.0% Elastane	1
290585	Lilac with White Spot Woven Fabric	21	65% Polyester; 35% Cotton	64.0% Polyester; 36.0% Cotton	1
290586	Pink with White Spot Woven Fabric	22	65% Polyester; 35% Cotton	64.4% Polyester; 35.6% Cotton	0.6
290587	Peach Floral Woven Fabric	23	65% Polyester; 35% Cotton	63.6% Polyester; 36.4% Cotton	1.4
290588	Black/Multi-Print Woven Fabric	24	65% Polyester; 35% Cotton	66.7% Polyester; 33.3% Cotton	1.7
290589	Green Christmas Party Woven Fabric	25	65% Polyester; 35% Cotton	63.7% Polyester; 36.3% Cotton	1.3
290590	Blue/White Zig Zag Woven Fabric	26	65% Polyester; 35% Cotton	65.5% Polyester; 34.5% Cotton	0.5
290591	Emerald Green Woven Fabric	27	65% Polyester; 35% Cotton	65.2% Polyester; 34.8% Cotton	0.2
290644	Cat Print Woven Fabric	28	65% Polyester; 35% Cotton	65.6% Polyester; 34.4% Cotton	0.6
290645	Black Bow Print Woven Fabric	29	65% Polyester; 35% Cotton	64.7% Polyester; 35.3% Cotton	0.3
290646	Blue with Pink Floral Print Woven Fabric	30	65% Polyester; 35% Cotton	67.3% Polyester; 32.7% Cotton	2.3
290592	Brown/White Check Woven Fabric	31	65% Cotton; 35% Polyester	52.5% Polyester; 47.5% Cotton	12.5
290593	Green/Blue Tartan Woven Fabric	32	65% Cotton; 35% Polyester	80.0% Polyester; 20.0% Cotton	15
290594	Brown/Black Tiger Print Woven Fabric	33	65% Cotton; 35% Polyester	67.2% Polyester; 32.8% Cotton	2.2
290595	Snow Leopard Woven Fabric	34	65% Cotton; 35% Polyester	68.3% Polyester; 31.7% Cotton	3.2
290596	Black/White Zebra Woven Fabric	35	65% Cotton; 35% Polyester	77.5% Polyester; 22.5% Cotton	12.5
290597	Camouflage Multi-Woven Fabric	36	65% Cotton; 35% Polyester	81.1% Polyester; 18.9% Cotton	16.1
290598	Wild Cat Printed Woven Fabric	37	65% Cotton; 35% Polyester	80.5% Polyester; 19.5% Cotton	15.5
290599	Red Tartan Woven Fabric	38	65% Cotton; 35% Polyester	78.6% Polyester; 21.4% Cotton	13.6
290647	Cow Print Woven Fabric	39	65% Cotton; 35% Polyester	79.2% Polyester; 20.8% Cotton	14.2
290648	Patchwork Print Woven Fabric	40	65% Cotton; 35% Polyester	67.5% Polyester; 32.5% Cotton	2.5
291042	Coral Woven Fabric	41	80% Polyester; 20% Cotton	82.8% Polyester; 17.2% Cotton	2.8
291045	Mint Woven Fabric	42	80% Polyester; 20% Cotton	97.2% Polyester; 2.8% Cotton	17.2
291046	Dinosaur Print Woven Fabric	43	80% Polyester; 20% Cotton	80.7% Polyester; 19.3% Cotton	0.7
291047	Ghost Print Woven Fabric	44	80% Polyester; 20% Cotton	82.0% Polyester; 18.0% Cotton	2
291284	Fox Print Woven Fabric	45	80% Polyester; 20% Cotton	90.4% Polyester; 9.6% Cotton	10.4
291529	Pink Floral Woven Fabric	46	80% Polyester; 20% Cotton	79.4% Polyester; 20.6% Cotton	0.6

**Table 2:** Composition error ranges with respect to the percentage of the sample population.

Error Range	Percentage of Population
<1%	30.4
≥1%	69.6
≥2%	50.0
≥3%	31.4
≥4%	23.9
≥5%	21.7
>10%	19.6

**Table 3:** Additional sample list provided by UK retailer.

Job No.	Fabric Description	Sample	Claimed Composition	Laboratory Test Results
901-0576	Ribbed Hat (Red)	TA01	60% Polyester, 26% Acrylic, 12% Nylon, and 2% Elastane	60.5% Polyester, 25.9% Acrylic, 12% Nylon, and 1.6% Elastane
129-5184	Pom Pom Hat (Grey)	TA02	56% Polyester; 44% Acrylic	55% Polyester; 45% Acrylic
661-7367	Quarter Zip Jumper (Black)	TA03	46% Polyester, 32% Viscose, and 22% Polyamide	46.1% Polyester, 32.2% Viscose, and 21.7% Polyamide
903-0298	Zip Hoody (Grey)	TA04	64% Cotton; 36% Polyester	64.4% Cotton; 35.6% Polyester
901-1373	Scarface Print T-shirt (Grey/Print)	TA05	99% Cotton; 1% Viscose	98.9% Cotton; 1.1% Viscose
450-9038	Long Sleeve T-shirt (Grey)	TA06	90% Cotton, 5% Elastane, and 5% Viscose	88.1% Cotton, 6.9% Elastane, and 5% Viscose
902-1905	Vertical Stripe Dress (Pink/White)	TA07	55% Linen; 45% Viscose	53.5% Linen; 46.5% Viscose
905-1894	Puff Sleeve Top (Blue)	TA08	98% Polyester; 2% Elastane	99% Polyester; 1% Elastane
902-2966	Stripe Dress (Green/White)	TA09	100% Cotton	100% Cotton
903-0461	Utility Trousers (Blue)	TA10	100% Lyocell	100% Lyocell
901-0205	Midi Skirt (Blue/Black)	TA11	95% Polyester; 5% Elastane	92.5% Polyester; 7.5% Elastane
123-1271	Stripe Mesh Slip (Cream)	TA12	Main 84% Nylon; 16% Elastane	83.6% Nylon; 16.4% Elastane
196-1666	Soft Touch Leggings (Black)	TA13	95% Viscose; 5% Elastane	95% Viscose; 5% Elastane
902-7032	Compression Shorts (Grey)	TA14	89% Nylon, 10% Elastane, and 1% Cotton	89% Nylon, 10% Elastane, and 1% Cotton
616-9546	Children’s Leggings (Blue)	TA15	95% Cotton; 5% Elastane	95% Cotton; 5% Elastane
616-9546	Children’s Leggings (Grey)	TA16	93% Cotton, 5% Elastane, and 2% Polyester	93% Cotton, 4.6% Elastane, and 1.9% Polyester
904-5074	Children’s Chinos (Brown)	TA17	98% Cotton; 2% Elastane	97.3% Cotton; 2.7% Elastane
100-5845	Quarter Button Jumper (Grey)	TA18	83% Cotton; 17% Nylon	81.9% Cotton; 18.1% Nylon
616-9546	Children’s Leggings (Multi)	TA19	95% Cotton; 5% Elastane	95% Cotton; 5% Elastane

**Table 4:** Independent test set results. Verified ground-truth and average model outputs are reported per region. TA19B is the only region with carbon-black dye and was correctly identified.

Sample	Ground Truth				Detection			
	Cotton	Polyester	Elastane	Black Dye	Cotton	Polyester	Elastane	Black Dye
TA04	0.64	0.36	0.00	0.00	0.70	0.26	0.01	0.00
TA08	0.00	0.99	0.01	0.00	0.02	0.97	0.03	0.00
TA09	1.00	0.00	0.00	0.00	0.96	0.03	0.04	0.00
TA11	0.00	0.93	0.07	0.00	0.04	0.95	0.04	0.00
TA15	0.95	0.00	0.05	0.00	0.96	0.02	0.05	0.00
TA16	0.93	0.02	0.05	0.00	0.95	0.03	0.03	0.00
TA17	0.97	0.00	0.03	0.00	0.96	0.02	0.05	0.00
TA19	0.95	0.00	0.05	0.00	0.96	0.02	0.05	0.00
TA19B	0.95	0.00	0.05	1.00	0.21	0.79	0.00	1.00

**Table 5:** Mean absolute errors (MAEs) for cotton, polyester, and elastane across all regions, non-black regions, and carbon-black dyed regions (standard deviation in parentheses).

	Cotton	Polyester	Elastane	Average
Mean error	3.8 (3.7)%	3.7 (3.6)%	0.9 (1.2)%	2.8 (3.3)%
Mean error (not-black region)	2.1 (1.6)%	2.0 (1.4)%	1.2 (1.0)%	1.8 (1.4)%
Mean error (carbon-black region)	7.1 (4.4)%	7.1 (4.0)%	0.4 (1.3)%	4.9 (4.7)%

## Data Availability

The original contributions presented in this study are included in the article. Further inquiries can be directed to the corresponding author.
